# The GTN patch: a simple and effective new approach to cardioprotection?

**DOI:** 10.1007/s00395-018-0681-2

**Published:** 2018-04-17

**Authors:** Derek M. Yellon, Zhenhe He, Rayomand Khambata, Amrita Ahluwalia, Sean M. Davidson

**Affiliations:** 1 The Hatter Cardiovascular Institute, University College London, 67 Chenies Mews, London, WC1E 6HX UK; 2 William Harvey Research Institute, Barts and The London Medical School, Queen Mary, University of London, Charterhouse Square, London, EC1M 6BQ UK

**Keywords:** Nitrate, Nitric oxide, Cardioprotection, Mouse, Ischaemia, Reperfusion

## Abstract

There remains a significant un-met need to reduce the extent of myocardial injury caused by ischaemia and reperfusion injury in patients experiencing an ST-elevation MI. Although nitric oxide is central to many cardioprotective strategies currently undergoing investigation, cardioprotection from the delivery of nitrates/nitrites has been inconsistently observed. The route of administration appears to be a critical variable. The glyceryl trinitrate (GTN) patch is commonly used as a simple and practical means of delivering nitric oxide to patients with ischaemic heart disease, but whether acute cardioprotection can be achieved by application of a GTN patch has not been investigated before. Here, we use a mouse model to demonstrate that a GTN patch is highly cardioprotective when applied immediately prior to 40 min occlusion of the left anterior coronary artery followed by 2 h reperfusion, reducing infarct size from 54 ± 4% in control mice, to 28 ± 4% (*P* < 0.001, *N* = 7). The degree of protection was similar to that achieved with a standard remote ischaemic preconditioning protocol. Furthermore, and of greater potential clinical relevance, a GTN patch was also protective when applied well after the initiation of ischaemia and 15 min prior to reperfusion (28 ± 4 vs 59 ± 4%; *P* < 0.01, *N* = 5). Confirmatory experiments verified the expected effect increase in plasma nitrite levels and decrease in blood pressure. The simplicity and rapidity of GTN patch application (easily applied in an ambulance or cardiac catheterization laboratory), and low cost (potentially relevant to low-income countries), make it attractive for further investigation.

## Introduction

There remains a significant un-met need to reduce the extent of myocardial injury caused by ischaemia and reperfusion injury in patients experiencing an ST-elevation MI (STEMI) [11–13, 15]. The ideal cardioprotective modality would be simple, effective, and able to be easily and rapidly delivered by the first responder. One promising procedure that is being investigated at present is remote ischaemic preconditioning (RIPC), in which several brief episodes of ischaemia and reperfusion are applied to a limb, thereby signalling to the heart to stimulate cardioprotection. RIPC has shown promise in reducing infarct size in STEMI patients in numerous proof of concept studies and is now undergoing investigation in a large, multi-centre, randomized trial [3, 14, 28]. However, the application of RIPC takes at least 30 min. Although the precise mechanism of RIPC is still under investigation, circulating nitrite originating from RIPC limb is believed to contribute to its cardioprotection [29, 36].

Organic nitrates, such as glyceryl trinitrate (GTN), are a highly effective means of rapidly delivering nitrate and nitrite into the blood stream, and are potent vasodilators. The transdermal GTN patch is widely used to reduce angina in patients with acute and chronic ischaemic syndromes due to coronary artery disease. Application of a GTN patch has been shown to induce delayed cardioprotection when applied to rabbits 72 h prior to infarction [19]. However, whether a GTN patch is protective when applied at a more clinically relevant time-point, such as during ischaemia, has never been investigated. We have investigated this hypothesis using an in vivo mouse model of ischaemia and reperfusion.

## Materials and methods

All animals received humane care in accordance with the United Kingdom Home Office Guide on the Operation of Animal (Scientific Procedures) Act of 1986. The investigation conforms to the guidelines from Directive 2010/63/EU of the European Parliament on the protection of animals used for scientific purposes or the NIH guidelines. All experiments were approved by the appropriate ethics committee and have, therefore, been performed in accordance with the ethical standards laid down in the 1964 Declaration of Helsinki and its later amendments.

10- to 12-week-old C57Bl/6 male mice were anaesthetized by i.p. injection of 80 mg/kg pentobarbitone at a concentration of 20 mg/ml in 0.9% (w/v) saline and maintained at 36.5 ± 0.5 °C on a heating mat. Surgery was started once pedal and tail reflexes were abolished and depth of anaesthesia was monitored throughout. Mice were intubated using a 19G cannula and ventilated using a MiniVent, type 845, Small Animal Ventilator (Harvard Apparatus, Kent, UK), supplemented with either room air or 100% oxygen, at a flow rate of 1.0 l/min with 2 cmH_2_O PEEP, stroke volume 200 µl at 130 strokes/min. After orotracheal intubation, the left common carotid artery running parallel to the trachea was carefully dissected and isolated, taking care not to damage the vagus nerve. The artery was cannulated using a thinned tip polyethylene tube (OD 0.96 mm) that was pre-filled with saline containing 10 units of heparin and was connected a calibrated pressure transducer. Mean arterial blood pressure was recorded using Labchart software.

A transdermal 5 mg GTN patch for human use was cut into eight equal pieces, and a single piece (containing 0.6 mg GTN) was applied to the depilated abdomen of an anaesthetized mouse. Inactive tape was applied to control mice. The change in blood pressure was measured after 10 min. The blood was then immediately removed by cardiac puncture, directly into a syringe containing sodium citrate. Plasma was obtained by centrifugation at 13,000×*g* for 15 min. Blood plasma was used for determination of total nitrate and nitrite (NO_*x*_) concentrations using ozone chemiluminescence. Plasma was filtered using Sartorius Vivaspin 500 3000 MWCO PES (Sartorius Stedim Biotech, Germany) at 4 °C, 14,000*g* for 60 min. Prior to use, filters were washed twice with low NO_*x*_ containing 18 MΩ dH_2_O. An NO analyser (NOA 280A, Sievers, UK) was used to measure NO based on the gas-phase chemiluminescent reaction between NO and ozone. To determine total NO_*x*_ concentration, samples were added to the purge vessel containing 0.1 M vanadium(III) chloride in 1 M hydrochloric acid refluxing at 95 °C under nitrogen. Nitrite concentration was determined by addition of samples to the purge vessel containing 0.09 M potassium iodide in glacial acetic acid under nitrogen at room temperature. Both of these conditions result in NO generation in the gas phase which is carried from the purge vessel to the NOA analyser where it reacts with ozone to emit a photon of light which is detected by the analyser. Nitrate concentration was calculated by subtraction of the nitrite concentration from the total NO_*x*_ [4, 20].

In a second set of experiments, the left anterior descending (LAD) coronary artery of anaesthetized mice was occluded (verified by ST elevation, hypokinesia and pallor) for 40 min followed by 2 h reperfusion, after which infarct size was measured by tetrazolium staining and expressed as a percentage of area at risk, determined using Evan’s blue. A GTN patch (1/8) or inactive tape was applied 10 min prior to ischaemia and left throughout the experiment. Remote ischaemic preconditioning (RIPC) was induced using a 6-mm lumen custom vascular occluder (Kent Scientific, CT, USA) around the right hind limb inflated to 250 mmHg to induce three cycles of 5 min ischaemia followed by 5 min reperfusion.

In a third set of experiments, ischaemia and reperfusion was performed as described above, but the patch (1/8) was applied after 25 min ischaemia, 15 min prior to reperfusion. All mice (i.e.: both control and those receiving the GTN patch) were transfused via the jugular vein with 250 µl blood from a donor mouse during reperfusion, in order to ensure blood pressure was maintained.

The results are shown as mean ± standard deviation of the mean. Statistical comparison of the groups was made by two-way ANOVA, with Bonferroni correction for multiple comparisons. A significance value of *P* < 0.05 was considered significant.

## Results

**Fig. 1:**
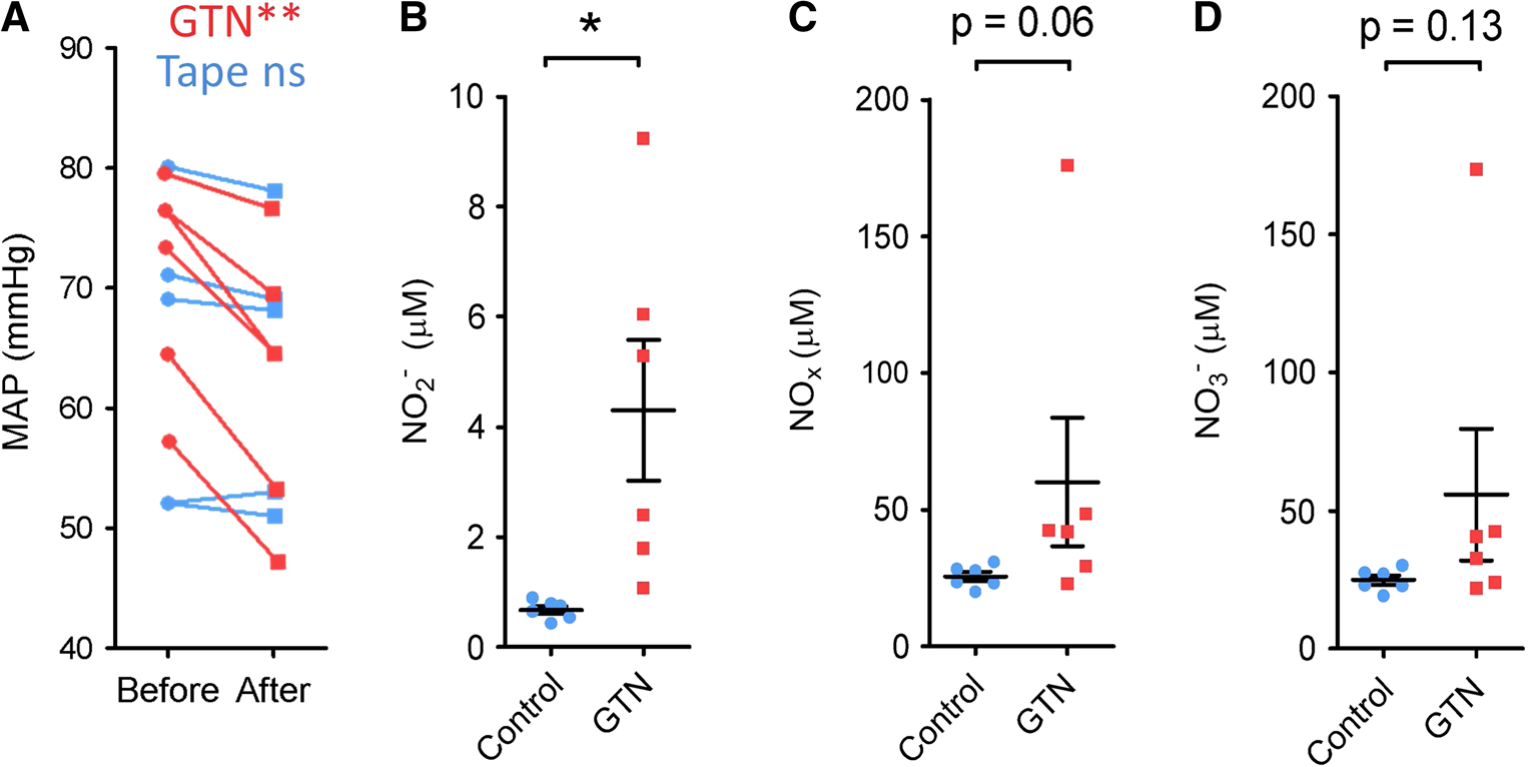
**a** The mean arterial blood pressure of anaesthetized mice decreased significantly 10 min after adhesion of a GTN patch to the abdomen (red), but not after control adhesive tape (blue). **P < 0.01 by paired *T* test. **b**–**d** In mice treated with a GTN patch, plasma nitrite (NO_2_^−^) concentration was significantly elevated after 10 min (*P < 0.05 by unpaired *T* test). The difference in plasma nitrate (NO_3_^−^) and total nitrates and nitrites was not significant

In anaesthetized mice, application of the GTN patch resulted in a 13 ± 2% decrease in mean arterial blood pressure (MAP) after 10 min (*P* < 0.01, *N* = 7) (Fig. 1a). Plasma nitrite levels were confirmed to be 6 ± 2-fold greater than in mice receiving the control tape (*P* < 0.05, *N* = 6-7) (Fig. 1b). Plasma nitrate and total nitrate + nitrite levels were similarly increased, although the difference was not significant (Fig. 1c, d).

**Fig. 2:**
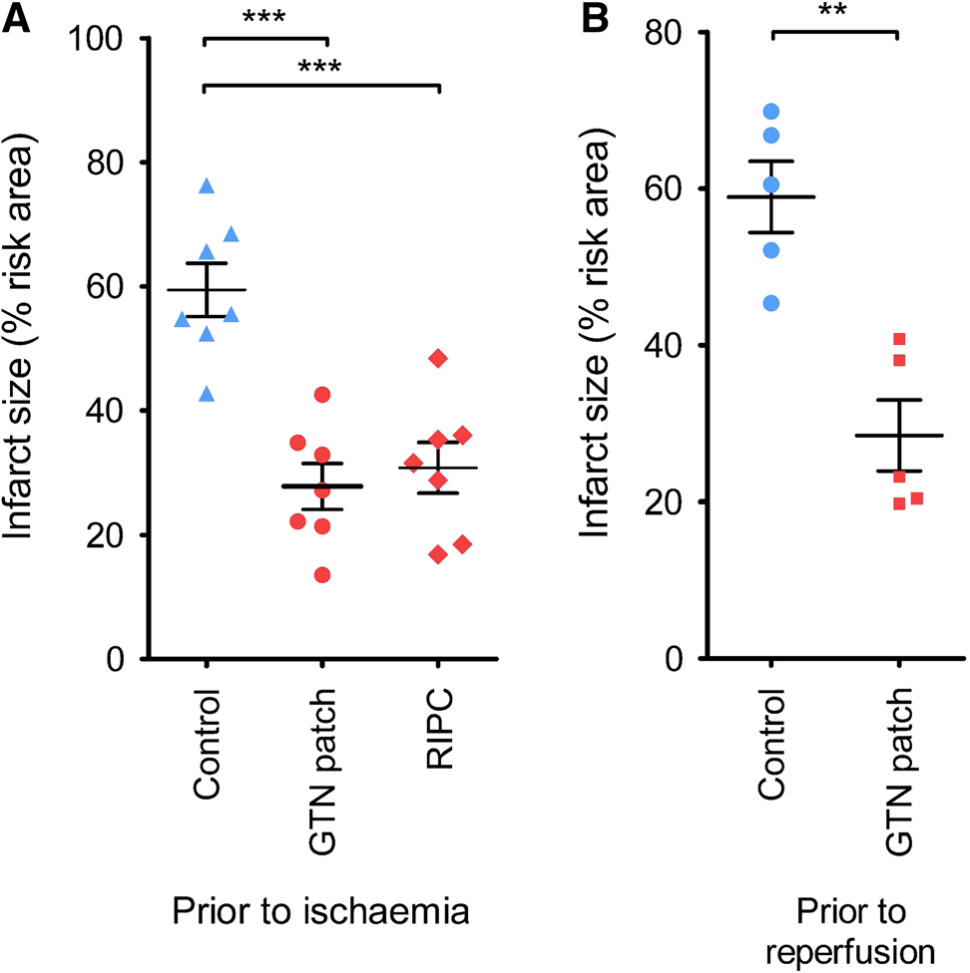
**a** Infarct size as a percentage of the area at risk was significantly decreased by a GTN patch applied 10 min prior to ischaemia. A positive control of remote ischaemic preconditioning was equally protective (RIPC). **b** GTN patch applied 15 min prior to reperfusion was cardioprotective. ***P < 0.001 one-way ANOVA and Tukey post-test. **P < 0.01 by unpaired *T* test

In a second set of experiments, application of a GTN patch 10 min prior to 40 min ischaemia followed by 2 h reperfusion significantly reduced myocardial infarct size from 54 ± 4 to 28 ± 4% (*P* < 0.001, *N* = 7) (Fig. 2a). The degree of protection was equivalent to a positive control consisting of a standard remote ischaemic preconditioning protocol of three cycles of 5 min hind-limb, blood-flow occlusion (Fig. 2a). It was important to verify that the GTN patch would reduce infarct size when applied prior to reperfusion, as this better reflects the potential scenario in which it would be applied to a patient in an ambulance. Application of the GTN patch 15 min prior to reperfusion was equally effective at reducing infarct size (28 ± 4 vs 59 ± 4%; *P* < 0.01, *N* = 5) (Fig. 2b).

## Discussion

These results suggest that the simple application of a transdermal GTN patch may be an effective means of protecting the heart against ischaemia and reperfusion injury, whether it is applied prior to ischaemia, or prior to reperfusion. Organic nitrates such as GTN are rapidly metabolized into nitrite, which is then reduced to the active vasodilator molecule, nitric oxide. Although nitric oxide is central to many cardioprotective strategies, including remote preconditioning, cardioprotection from the delivery of nitrates/nitrites has been inconsistently observed. The route and timing of administration appears to be a critical variable [19, 31]. For example, i.v. infusion of sodium nitrite at the time of reperfusion did not reproducibly protect animals in the CAESAR trial, but intracoronary or oral nitrite has successfully reduced infarct size in other animal studies [7, 16, 32, 36]. A recent systematic review of nitric oxide therapies given at reperfusion, which included 21 animal studies, found significant protection in the majority of studies, with a mean overall reduction in infarct size of 17.93% (95% confidence interval: 22.05, 13.81) [2]. The exceptions in which no protection was observed included 5 studies: two in which 2 µg/kg/min GTN was administered to rabbits or pigs via continuous IV infusion starting 5 or 10 min prior to reperfusion [25, 30]; a study in rats in which 4 mg/kg NaNO_2_ was administered IV starting 10 s after the onset of reperfusion [1]; a study from 1997 in which acidified NaNO_2_ was infused IV in adult male mongrel dogs starting at the time of reperfusion [37]; and a study in which inhalation of 80 ppm NO was initiated 0.5 min prior to reperfusion in mice [27].

In humans, a 4-h intravenous infusion of GTN protects the heart against ischaemia during coronary angioplasty conducted 24 h later, as evidenced by reduced ST-segment elevation, ischaemic dysfunction, and pain [24]. However, how well these clinical endpoints relate to experimentally used endpoints of myocardial damage caused by ischaemia and reperfusion injury, and the precise relationship between GTN and preconditioning are not entirely clear [18]. Several studies of STEMI patients from the thrombolytic era, as well as a more recent study, have not found evidence for infarct size reduction after nitrate or nitrite infusion [2]. Interestingly, in a recent study, intracoronary but not i.v. infusion of nitrites reduced infarct size in STEMI patients with completely occluded arteries at admission [21, 33], in whom reperfusion injury might be expected to be a significant factor. GTN is sometimes administered to patients undergoing surgical coronary revascularization, at the discretion of the anaesthesiologist. However, when comparing patients who do not receive GTN to those who receive 0.042 ± 0.024 mg/kg GTN, no evidence for a reduction peri-procedural injury was found, as determined by cTnI release [23]. Surprisingly, the simple, practical, and cost-effective method of inducing acute cardioprotection by simply applying a GTN patch prior to reperfusion had not been previously investigated.

Nitrates and nitric oxide can protect the heart via multiple possible mechanisms, but this appears to be independent from their vasodilatory effects [5]. Nitric oxide can directly nitrosate proteins and also activate a pathway involving nitric oxide-sensitive guanylyl cyclase and cGMP-dependent protein kinase type I (PKGI) in cardiomyocytes [6, 9]. Recently, these have been shown to activate cardioprotection via large-conductance, Ca^2+^-dependent potassium (BK) channels [8, 17].

The GTN patch used here was designed to deliver 5 mg/day, so the 1/8 patch we used is expected to deliver 0.026 mg/h. However, we did not perform a dose–response experiment to establish the optimal dose of GTN. Basal levels of plasma nitrite vary considerably and decrease with increasing numbers of cardiovascular risk factors [22]. Plasma nitrates are also affected by dietary nitrates. Dietary inorganic nitrate ingestion or supplementation causes a dose-dependent elevation in plasma nitrite concentration with a consequent decrease in blood pressure in healthy volunteers. A meta-analysis found a significant association of the dose of inorganic nitrate supplementation with decline in systolic blood pressure, with a factor of − 0.12 mmHg/1.0 mmol nitrate (*P* < 0.05) [34]. Although it would be of interest to test for an association between infarct size and plasma nitrite, this was not possible in our study since the quantity of blood necessary for the nitrite assay made it impossible to perform infarction using the same mice.

When using this approach, it is important to be aware that excessive nitrate concentrations are potentially damaging, and in addition to nitrate tolerance, can cause nitro-oxidative stress. Endothelial dysfunction, formation of DNA adducts, and even apoptotic death of vascular cells has been seen when 10–50 mg/kg/day nitrate was administered to rats for 3 days [26]. Furthermore, prolonged exposure to GTN can induce tolerance. For example, 7 days of transdermal GTN exposure abolished pacing-induced preconditioning in conscious rabbits [35] and abrogated the effectiveness of remote ischaemic conditioning in rat myocardium and human volunteers [10]. Interestingly, however, direct myocardial protection with glyceryl trinitrate (GTN) may be preserved in the state of vascular nitrate tolerance [5].

Clinical studies are required to establish the potential efficacy of using the transdermal GTN patch in the setting of patients who are experiencing an STEMI. These will necessitate careful determination of the appropriate dose and timing of administration. However, its simplicity and rapidity (easily applied in an ambulance or cardiac catheterization laboratory), and low cost (potentially relevant to low-income countries), make it attractive for further investigation.
